# Telmisartan Modulates Glial Activation: *In Vitro* and *In Vivo* Studies

**DOI:** 10.1371/journal.pone.0155823

**Published:** 2016-05-17

**Authors:** Nofar Torika, Keren Asraf, Abraham Danon, Ron N. Apte, Sigal Fleisher-Berkovich

**Affiliations:** 1 Department of Clinical Biochemistry and Pharmacology, Ben-Gurion University of the Negev, Beer-Sheva P.O.B 653, Israel; 2 Department of Microbiology and Immunology, Ben-Gurion University of the Negev, Beer-Sheva P.O.B 653, Israel; Max-Delbrück Center for Molecular Medicine (MDC), GERMANY

## Abstract

The circulating renin-angiotensin system (RAS), including the biologically active angiotensin II, is a fundamental regulatory mechanism of blood pressure conserved through evolution. Angiotensin II components of the RAS have also been identified in the brain. In addition to pro-inflammatory cytokines, neuromodulators, such as angiotensin II can induce (through angiotensin type 1 receptor (AT_1_R)) some of the inflammatory actions of brain glial cells and influence brain inflammation. Moreover, in Alzheimer’s disease (AD) models, where neuroinflammation occurs, increased levels of cortical AT_1_Rs have been shown. Still, the precise role of RAS in neuroinflammation is not completely clear. The overall aim of the present study was to elucidate the role of RAS in the modulation of glial functions and AD pathology. To reach this goal, the specific aims of the present study were a. to investigate the long term effect of telmisartan (AT_1_R blocker) on tumor necrosis factor-α (TNF-α), interleukin 1-β (IL1-β) and nitric oxide (NO) release from glial cells. b. to examine the effect of intranasally administered telmisartan on amyloid burden and microglial activation in 5X familial AD (5XFAD) mice. Telmisartan effects *in vivo* were compared to those of perindopril (angiotensin converting enzyme inhibitor). Long-term-exposure of BV2 microglia to telmisartan significantly decreased lipopolysaccharide (LPS) -induced NO, inducible NO synthase, TNF-α and IL1-β synthesis. The effect of Telmisartan on NO production in BV2 cells was confirmed also in primary neonatal rat glial cells. Intranasal administration of telmisartan (1 mg/kg/day) for up to two months significantly reduced amyloid burden and CD11b expression (a marker for microglia) both in the cortex and hipoccampus of 5XFAD. Based on the current view of RAS and our data, showing reduced amyloid burden and glial activation in the brains of 5XFAD transgenic mice, one may envision potential intervention with the progression of glial activation and AD by using AT_1_R blockers.

## Introduction

The pathology of Alzheimer's disease (AD) is associated with neuroinflammation, which involves activation of central resident immune cells (e.g., microglia, astrocytes) and infiltrating peripheral macrophages [1]. Microglia are known as the macrophages of the brain, representing the first immune response defense in the central nervous system (CNS), and, therefore, can be rapidly activated in response to any central pathological changes [2]. Microglial activation plays a dual role in neurodegeneration, since microglia can aid the repair and regeneration of neurons, but they can also instigate neuronal damage [3]. The brain inflammation involved in AD pathology includes the deposition of amyloid β (Aβ) in areas where pronounced microglia and astrocytes cells activation is observed [4,5].

Glial activation by Aβ results in the production of various pro-inflammatory cytokines, prostaglandins and nitric oxide (NO), which contribute to synaptic impairment and neuronal damage [3,4,6]. The cellular injury promotes additional inflammatory cascades, increases cellular injury, and aggravates AD pathology [3,6–10]. Several lines of evidence support the neurotoxic role of glia-secreted NO in neuronal death *in vitro* [11,12]. Glial inducible nitric oxide synthase (iNOS) isoform has been linked with neurodegeneration [13,14]. Elevated levels of tumor necrosis factor-α (TNF- α) have been also observed in the cerebrospinal fluid (CSF) and serum of AD patients [15]. Interleukin 1- β (IL1-β) levels were reported to increase in early onset AD patients' serum [16]. Also, both cytokines can aggravate brain inflammation by sustaining microglial activation [17,18].

Accumulating evidence indicate that the renin-angiotensin system (RAS) may contribute to the brain inflammation associated with AD pathology [19]. An intrinsic brain RAS, which is distinct from the peripheral one, was identified [20]. The peripheral hormone and neuropeptide angiotensin II (AngII) is considered the major RAS effector, and acts mainly via stimulation of the angiotensin type 1 receptor (AT_1_R) [21]. The latter may serve as neuroinflammatory instigator [7,22]. AngII is formed upon the hydrolysis of angiotensin I by the angiotensin converting enzyme (ACE). Angiotensinogen, the angiotensin I precursor, is abundant in the brain’s extracellular and cerebrospinal fluids and is mostly produced in glial cells (mainly by astrocytes and, to a lesser extent, by microglia) [23–25]. Receptors for AngII have been identified in rat, monkey, and human glial cells (microglia and astrocytes) [24,26,27]. Hyperactivation of brain AT_1_R promotes hypertension and vulnerability to ischemia and to vascular and tissue inflammation, and may enhance neuronal loss and neurodegeneration [7,22,28–30]. There is evidence indicating that RAS blockade by orally administered ACE inhibitors (ACEI) or AT_1_R blockers (ARBs), e.g perindopril or telmisartan respectively, may have a beneficial effect on cognitive functions in AD [31–36]. However, improved targeting the brain by these agents is still a major challenge [37]. Intranasal administration may offer a solution, as this noninvasive procedure can bypass the blood brain barrier (BBB) and allow direct entry to the CNS [37].

The overall aim of the present study was to elucidate the role of RAS in the modulation of glial functions and AD pathology. To reach this goal, the specific aims of the present study were a. to investigate the long term effect of telmisartan (ARB) on NO release from BV2 murine microglial cells as well as neonatal primary microglial cells. Regulation of TNF-α, IL1-β release and iNOS expression by telmisartan will be studied in BV2 cells. b. to examine the effect of intranasally administered telmisartan on amyloid burden and microglia/macrophages activation in Alzheimer’s disease (AD) mice. Telmisartan effects *in vivo* were compared to those of perindopril (ACEI).

The five familial Alzheimer’s disease (5XFAD) transgenic mouse model was used. This model expresses three mutations in the gene for the amyloid precursor protein (APP) and two mutations in the gene for presenilin 1 (PS1). These mutations, in turn, lead to early production of a 42-amino acids Aβ peptide, to brain inflammation, and to glial activation associated with Aβ plaques [38].

## Materials and Methods

### Cell culture

#### BV2 murine microglial cells

BV2 murine microglial cells were obtained from Professor Rosario Donato (Dep. of Experimental Medicine and Biochemical Sciences, University of Perugia, Italy) [39,40]. Cells were routinely maintained in RPMI-1640 medium with fetal bovine serum (10%), penicillin (100 U/ml), streptomycin (100 μg/ml)) and L-glutamine (4 mM) at 37°C in 5% CO2 humidified air. Prior to each experiment, cells were grown for 24 h on 24-well and 6-well plates at a concentration of 3×10^5^ and 1X10^6^ cells per well, respectively.

#### Primary rat neonatal glial cells and microglia

Whole brains of neonatal Wistar rats, 0–24 h of age were used in order to obtain rat primary glial cultures, according to previous reports [41,42]. Pups were sacrificed by decapitation, the meninges were removed and a steel mesh and nylon sieves of 60 μm pore size were used to harvest brain cells. The cells were seeded in poly-l-lysine- coated- 24-well plates and 75 cm2 flasks at a concentration of 1X10^6^ cells per well and 30-35X10^6^ cells per flask, respectively. The medium culture included high glucose DMEM with 10% heat inactivated FCS, penicillin (100 U/ml), streptomycin (100 μg/ml), l-glutamine (0.2 mM) and insulin (100 U/ml). Immunocytochemistry studies revealed that these cultures contain about 80% astrocytes and about 20% microglia (Levant 2006).

Isolated microglia were obtained from a mixed culture of astrocytes and microglia grown in flasks, at a 37°C humidified atmosphere with 5% CO_2_, for 12 days. Medium was replaced once a week after culturing. On day 12, flasks were shaken at 200 rpm for 1.5 h at 37°C and medium containing floating microglial cells used for cells reseeding in a 24-well plates, 1X10^6^ cells per well. Culture purification immunocytochemistry analysis indicated >95% microglia cells for this enriched microglia procedure [43].

Mixed cultures of glial cells were obtained from cells seeded on poly-l-lysine-coated 24-well plates and grown in a humidified incubator at 37°C with 5% CO2 for 21 days. Culture medium was replaced twice a week prior to experiments.

Serum- free medium (SFM) was added to the cells prior to each experiment. After 4 h of SFM incubation, cells were treated with SFM containing 0.1% BSA and HEPES buffer (10 mM), pH 7.4 in the presence or absence of test agents for the indicated periods of time.

Telmisartan was purchased from Tocris Biosience, Bristol UK and lipopolysaccharides (LPS) was from Sigma-Aldrich.

### Determination of NO levels (Griess reaction)

Culture supernatant nitrite levels, as an indicator for NO production, were determined by a colorimetric assay using Griess reagent (Sigma—Aldrich). A standard curve of sodium nitrite was used for nitrite concentrations. Equal volume (100 μl) of culture supernatant and Griess reagent were mixed in a 96-well plate and incubated at room temperature for 15 min (light avoided). Then, absorbance at 540 nm was measured using a microplate reader (model 680, Bio-Rad). Meanwhile, cells were harvested with 4°C SFM and counted using Z1 Coulter counter (Coulter Electronics, Miami, FL) [40].

### TNF-α and IL1-β protein assay by ELISA

TNF-α and IL1-β levels in the medium (supernatant) were assayed using enzymelinked immunosorbent assay (ELISA) kits (BD Biosciences, San Diego, CA) according to the manufacturer’s instructions.

### Western blot analysis

Whole cell lysates were separated on 7.5% polyacrylamide-SDS gels and transferred to nitrocellulose membranes. After blocking (4% BSA, 90 min at room temperature), membranes were incubated overnight at 4°C with specific rabbit anti-iNOS antibody (1:1000, Cayman Chemicals, USA). Following washing, the blots were incubated for 90 min at room temperature in the corresponding-conjugated donkey anti-rabbit antibody (1:10000, GE Healthcare, Buckinghamshire, UK). The position of the individual protein was detected using enhanced chemiluminescence (ECL) solution (according to the manufacturer’s instructions) followed by exposure to X-ray film (Fuji medical X-ray film, FujiFilm). Band intensity analysis was performed using a computerized image analysis system (EZ Quant-Gel 2.2, EZQuant Biology Software Solutions Ltd., Israel). Protein load was normalized by β-actin protein level measurements using mouse anti-β-actin antibody (1:4000, Sigma-Aldrich) followed by exposure to horseradish peroxidase-conjugated goat anti-mouse antibody (1:20,000, Jackson immunoReaserch Inc., USA).

### Mice

5XFAD transgenic mice (Tg6799), kindly provided by Professor Robert Vassar (Department of Cell and Molecular Biology, Northwestern University, Chicago, Illinois 60611), were used. These mice exhibit three familial Alzheimer’s disease (FAD) mutations in the human APP695 (Swedish K670N, M671L; Florida I716V and London V717I) and two mutations in the human presenilin-1 (PSEN-1) (M146L, L286V) under the transcriptional control of the neuron-specific mouse Thy-1 promoter [38]. Wild-type (WT) C57BL/6 mice were purchased from Harlan (Jerusalem, Israel) and bred with hemizygous transgenic mice (TG). Both male and female were used. Tail DNA was used for PCR genotyping analysis and detection of the human APP gene. The mice were housed in cages in an air-conditioned room with controlled temperature (22 ± 2°C) and humidity (65 ± 5%) and maintained on a 12h light/dark cycle. Mice were randomly divided into three groups (n = 6–8 animals each, both genders), telmisartan-treated (Tocris Biosience, Bristol UK) (1mg/kg/day) or perindopril-treated (Sigma-Aldrich) (1mg/kg/day) and vehicle-treated mice. The control group included wild-type mice treated with telmisartan or perindopril. Two-month-old mice were treated intranasally (total of 6 μl administered as one 3 μl drops to each nostril) every day for 3.5 to 8 weeks. No significant differences in body weights were observed between different mice groups during and at the end of the experiments.

All primary cell cultures and mice procedures were approved by the Institutional Animal Care and Use Committee of the Ben-Gurion University of the Negev (Beer Sheva, Israel; approval number IL-30-08-2011-15 and IL-54-08-2015-19) [44].

### Immunohistochemistry

A mixture of 0.1ml clorketam and 0.1ml sedaxylan was used to anesthetize mice through intra peritoneal (i.p) injection. Then, cardiac perfusion was performed with cold PBS. The brains were removed and one hemisphere of each was incubated in 4% paraformaldehyde (PFA) solution for 24h and transferred to 30% sucrose solution for 48h at 4°C. The tissue was then placed into molds containing OCT compound (Tissue-Tek, Torrance, CA, USA) and frozen at -80°C. Immunohistochemical staining was proformed on 40μm sagittal sections (cut by caryostat). First, the sections were rinsed in 0.05% PBS/Tween 20 solution and for further 30 min in 0.5% PBS/Triton X-100 solution. In order to prevent nonspecific binding, blocking was made using primary Ab diluting buffer (GBI labs, Bothell, WA). Immunohistochemical staining was performed for CD11b and Aβ proteins by rabbit anti human Aβ antibody (1:250, gift from Professor Alon Monsonego, The Shraga Segal Department of Microbiology and immunology, faculty of Health Sciences and the National institute of Biotechnology in the Negev, Ben-Gurion University, Beer-Sheva, Israel) and Rat anti mouse/human CD11b antibody (1:25, Biolegend). An additional rinsing by 0.05% PBS/Tween 20 solution was made, followed by secondary antibody incubation with Cy3-conjugated donkey anti rabbit IgG (1:1000, Jackson ImmunoResearch Laboratories, 711-165-152) or Alexa flour 488-conjugated goat anti rat IgG (1:250, Jackson ImmunoResearch Laboratories, 112-545-003), respectively. Finally, tissues were placed on charged slides, covered with mounting medium containing DAPI (Vector labs) and stored in 4°C [44].

### Confocal imaging analysis

Quantification analysis of Aβ plaques and CD11b positive cells in the brain was performed in sections (40 μm thick) per hemisphere stained for Aβ and CD11b. In each experiment five sections from each animal (from the cortex and the hippocampus) were analyzed. Fluorescence baseline intensity was first obtained in sections from control mice (wild type). All images were obtained using an Olympus FluoView FV1000 confocal microscope (Olympus, Hamburg, Germany) at a 1024 X 1024 pixel resolution with X10 objective. Aβ and CD11b staining was quantified using ImageJ software (version 1.40C, NIH, USA) with threshold function. The intensity threshold was set by marking areas stained only with CD11b or Aβ in the hippocampus and cortex. The average florescence stained area was calculated for each treated group [45].

### Statistical analysis

Experimental data arepresented as the mean±SEM. For significance assessment between groups, one-way analysis of variance (ANOVA) and post hoc multiple comparison test (Tukey—Kramer Multiple Comparison Test) were performed. Statistical significance was considered at P< 0.05.

## Results

### Telmisartan attenuates the expression of inflammatory markers in LPS-induced glial cells

**Fig 1:**
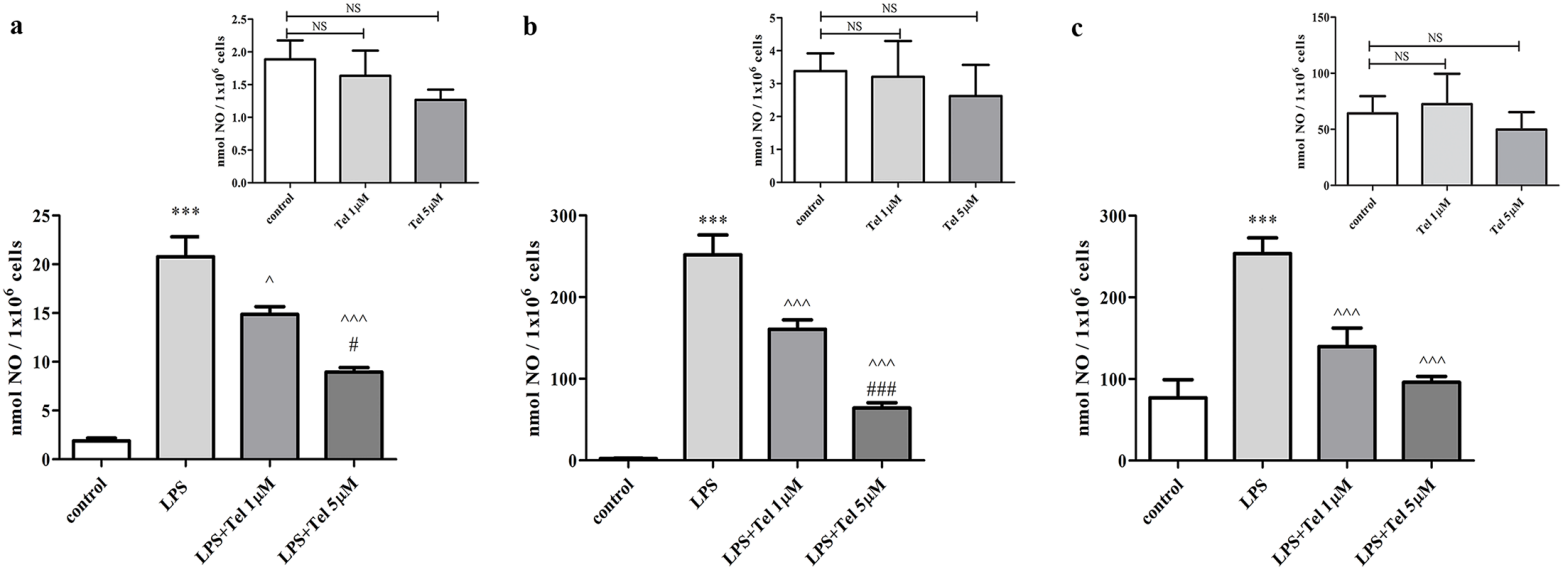
Telmisartan decreased NO production in LPS-stimulated BV2 and primary neonatal rat glial cells. BV2 microglia **(a)**, primary microglial cells **(b)** and mixed glial cells **(C)** were incubated with LPS (7 ng/ml for BV2 cells and 0.5 μg/ml for primary cultures) in the presence or absence of telmisartan (Tel), at 1 μM or 5 μM, for 24h. NO levels were determined in the media and normalized to cells number. *Insets*: NO levels measured in non-stimulated cells treated with Tel at 1 μM or 5 μM concentrations. Data are presented as means ± SEM and are representatives of 2–3 independent experiments (overall n = 8–12). Statistical significance was determined using one-way ANOVA, followed by a Tukey—Kramer Multiple Comparison Test. ***P < 0.001 vs. control (non-stimulated cells); ^P < 0.05 vs. LPS; ^^^P < 0.001 vs. LPS; ^#^P < 0.05 vs. LPS+Telmisartan 1μM; ^###^P < 0.001 vs. LPS+Telmisartan 1μM; NS (non-significant) vs. control.

We examined the production of NO in BV2 cells induced by LPS (7 ng/ml) and treated with telmisartan (Fig 1a). While LPS significantly increased NO production as compared to non-stimulated cells (control), telmisartan attenuated this effect in a concentration-dependent manner. Specifically, a 24h incubation with 1 μM or 5 μM telmisartan decreased LPS-induced NO production by 30% and 60%, respectively (Fig 1a). Telmisartan did not affect NO production in non-stimulated (control) cells (Fig 1a inset). The effect of telmisartan on NO production in BV2 cells was confirmed in primary neonatal rat microglia (Fig 1b) and mixed glial cultures (Fig 1c). In both cultures, LPS (0.5μg/ml) stimulated NO release to the media and telmisartan reduced NO production even more than in BV2 cells. In primary microglia, 1μM and 5μM telmisartan reduced NO production by 30% and 74%, respectively. In mixed glial cells, telmisartan attenuated NO release by 45% and 62%, respectively. In both types of primary glial cell cultures telmisartan did not alter basal levels of NO (Fig 1b and 1c insets).

**Fig 2:**
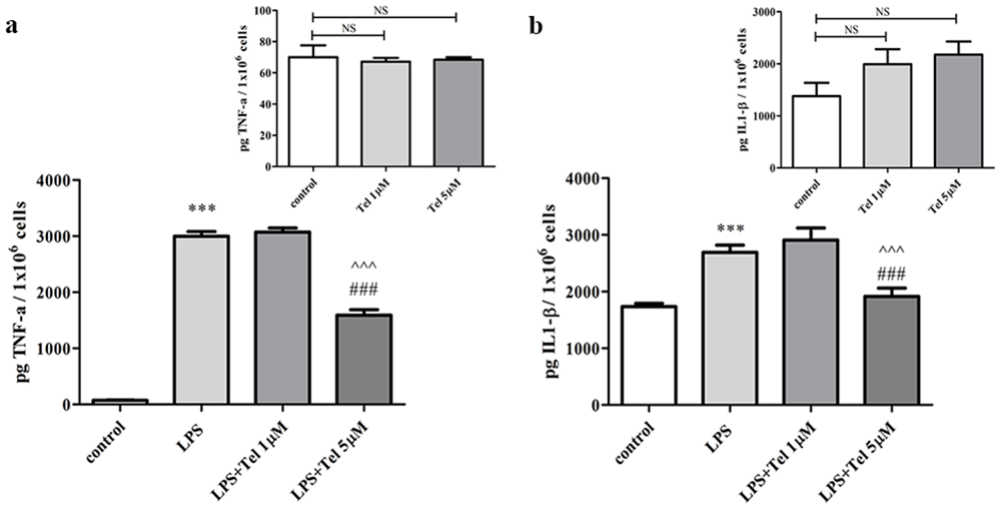
Telmisartan attenuated LPS-induced TNF-α and IL1-β release from BV2 microglia cells. Cells were incubated with LPS (7 ng/ml) in the presence or absence of telmisartan (Tel), at 1 μM or 5 μM, for 24h. Media were collected and analyzed for TNF-α and IL1-β levels and cells were counted. *Insets*: TNF-α and IL1-β levels measured in non-stimulated cells treated with Tel at 1 μM or 5 μM. Data presented as means ± SEM and are representatives of 2–3 independent experiments (overall n = 8–12). Statistical significance was determined using one-way ANOVA, followed by a Tukey—Kramer Multiple Comparison Test. ***P < 0.001 vs. control (non-stimulated cells); ^^^P < 0.001 vs. LPS; ^###^P < 0.001 vs. LPS+Telmisartan 1μM; NS (non-significant) vs. control.

Telmisartan (5 μM) also substantially attenuated the LPS-induced TNF-α and IL1-β production by approximately 50% and 30%, respectively (Fig 2a and 2b). However, it did not change TNF-α or IL1-β production in non-stimulated BV2 cells (Fig 2a and 2b insets).

**Fig 3:**
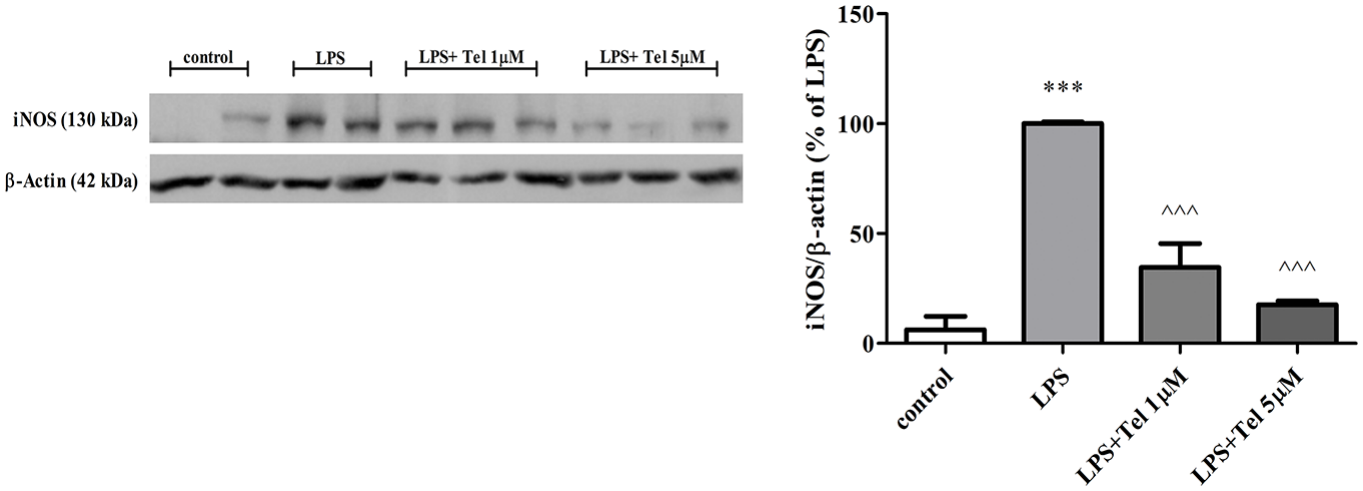
Telmisartan decreased iNOS expression in LPS-induced BV2 microglia. Cells were incubated with LPS (7 ng/ml) in the presence or absence of telmisartan (Tel), at 1 μM or 5 μM, for 24h. 40 μg protein of whole cell lysate was loaded on 7.5% polyacrylamide-SDS gels. Analysis of iNOS was performed using antibodies against iNOS (130 kDa) and β-actin (40 kDa). Results are representative of two independent experiments and are presented as means ± SEM (overall n = 4–6). ***P<0.001 vs. control; ^^^P<0.001 vs. LPS.

As shown in Fig 3, 24h exposure of BV2 cells to LPS (7 ng/ml) resulted in robust increase in iNOS protein levels, namely, by more than 90% as compared with the control. In the LPS-induced cells, 24h incubation with 1 μM or with 5 μM telmisartan significantly reduced iNOS expression levels by 65% and 85%, respectively.

### Intranasal administration of telmisartan decreased Aβ burden and CD11b staining in brains of 5XFAD mice

**Fig 4:**
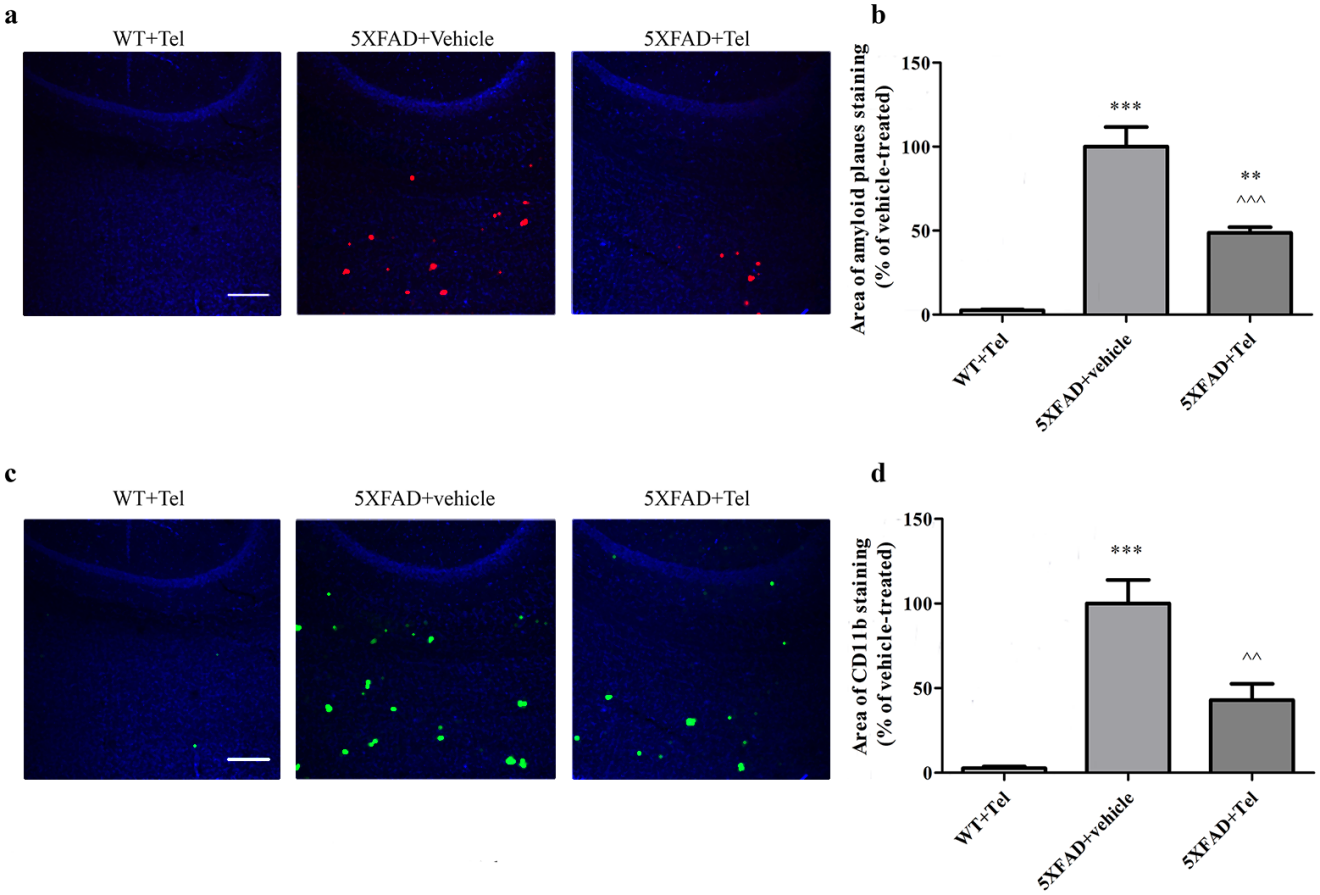
Intranasal administration of telmisartan decreases amyloid plaques and CD11b staining in the cortex of 3-month-old 5XFAD mice. Mice were treated with telmisartan (Tel) or with vehicle (N,N-dimethylformamide/polyethylene glycol 400/saline (2:6:2) [47]) for 3.5 weeks, and their brains were sectioned and immunolabeled with anti-Aβ (red) and anti-CD11b (green) antibodies and countersained with DAPI (blue). **(a, c)** Representative cortex brain section of WT or 5XFAD mice treated with 1 mg/kg/day telmisartan or with vehicle. Each experiment included 5 mice per group (n = 15 in total). (**b, d**) Quantification of the average sum of Aβ-stained area **(b)** and of CD11b-stained area **(d)**, represented as the mean ± SEM percentage of stained area in the corresponding vehicle-treated group, in at least 3 determinaions. Statistical significance was determined using one-way ANOVA, followed by a Tukey—Kramer Multiple Comparison Test. **P<0.01 vs. WT+Tel; ***P<0.001 vs. WT+Tel, ^^P<0.01 vs. 5XFAD+ vehicle; ^^^P<0.001 vs. 5XFAD + vehicle. **(e, f)** Representative hippocampal section of 5XFAD mice treated with vehicle. Scale bar is 200 μm.

**Fig 5:**
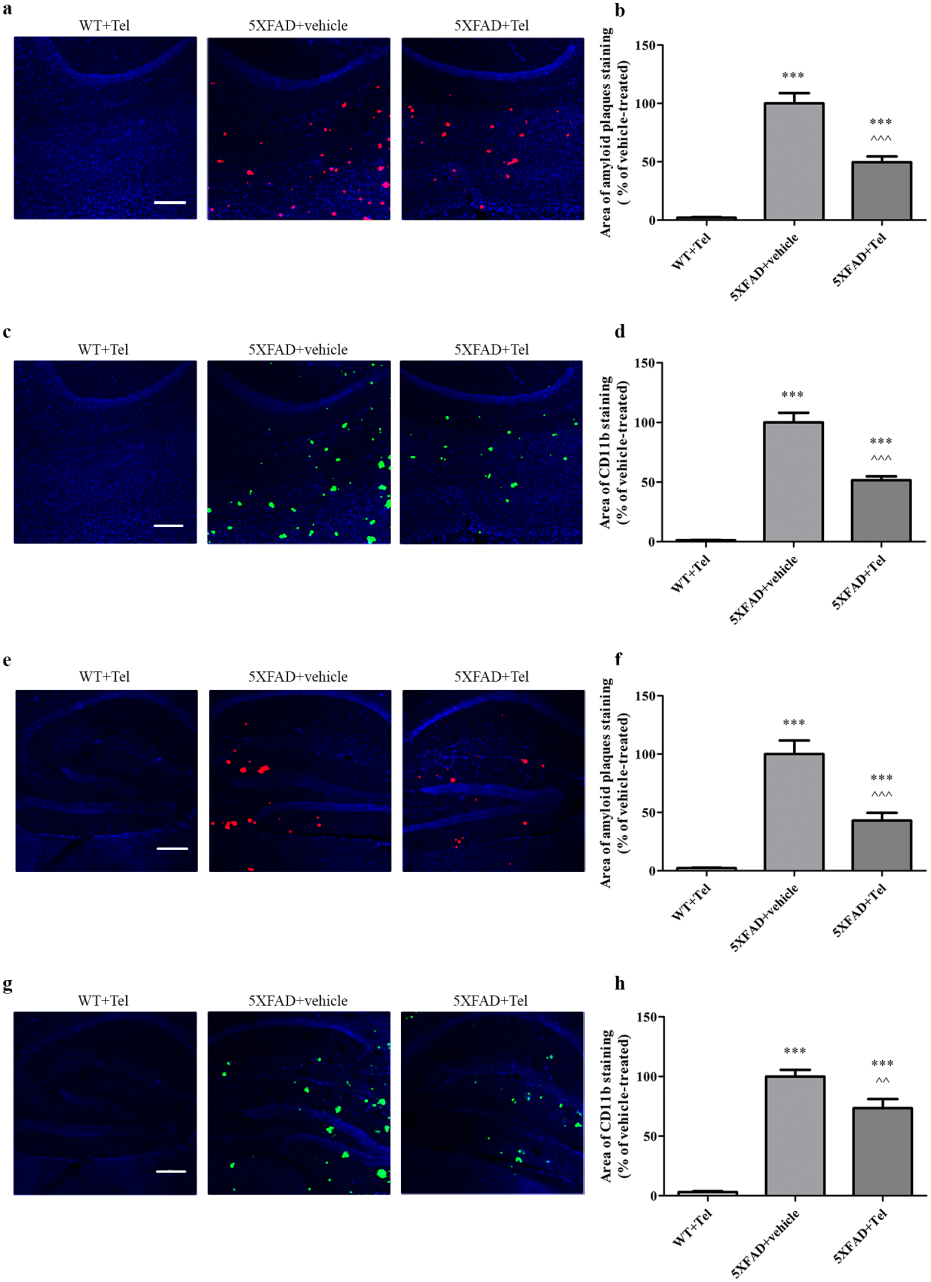
Intranasal administration of telmisartan decreases amyloid plaques and CD11b staining in the cortex and hippocampus of 4-month old 5XFAD mice. Mice were treated with telmisartan (Tel) or vehicle (N,N-dimethylformamide/polyethylene glycol 400/saline (2:6:2) [47]) for 8 weeks. The brains of 4-month-old mice were sectioned and immunolabeled with anti-Aβ (red) and anti-CD11b (green) antibodies and countersained with DAPI (blue). (**a, c**) Representative cortical sections from WT or 5XFAD mice treated with 1 mg/kg/day telmisartan or with vehicle. (**e, g**) Representative hippocampal sections of WT or 5XFAD mice treated with 1 mg/kg/day telmisartan or with vehicle. Each experiment included 6 mice per group (n = 18 in total). (**b, d, f, h**) Quantification of the average sum of Aβ-stained area **(b, f)** or of CD11b-stained area **(d, h)**, represented as the mean ± SEM percentage of stained area in the corresponding vehicle-treated group in at least 3 determinants. Statistical significance was determined using one-way ANOVA, followed by a Tukey—Kramer Multiple Comparison Test. ***P<0.001 vs. WT+Tel; ^^P<0.01 vs. 5XFAD+vehicle; ^^^P<0.001 vs. 5XFAD+vehicle. Scale bar is 200 μm.

As expected, the cortex of 3-month-old wild-type (WT) mice treated intranasally with telmisartan did not show any Aβ plaques or CD11b (indicative of microglia and macrophages activation [46]) staining, whereas both these parameters were significantly increased in age-matched 5XFAD mice treated intranasally with the vehicle (Fig 4). However, as compared with 5XFAD mice treated with the vehicle, age-matched 5XFAD mice treated intranasally for 3.5 weeks with 1 mg/kg/day telmisartan showed a ~52% and ~57% reduction in the cortical area covered by Aβ plaques (Fig 4a and 4b) and CD11b staining, respectively (Fig 4c and 4d). Prolonged (8 weeks) intranasal treatment with telmisartan (Fig 5) also attenuated amyloid plaques and CD11b by ~50% staining, both in the cortex (Fig 5a–5d) and in the hippocampus (~50% for Aβ and ~25% for CD11b; Fig 5e–5h). WT mice treated with telmisartan did not display any plaque deposition or CD11b staining in the hippocampus or in the cortex. The amount of amyloid plaque depositions and CD11b increased with age (compare Fig 4 with Fig 5). Plaque formation and gliosis appear mainly in the cortex but to a lesser extent in the hippocampus in immature stages of 5XFAD mice [38] therefore, the hippocampal section quantification is not shown for the short treatment of 3.5 weeks.

### Intranasal administration of perindopril reduces amyloid plaques burden and CD11b staining in the cortex of 5XFAD mice

**Fig 6:**
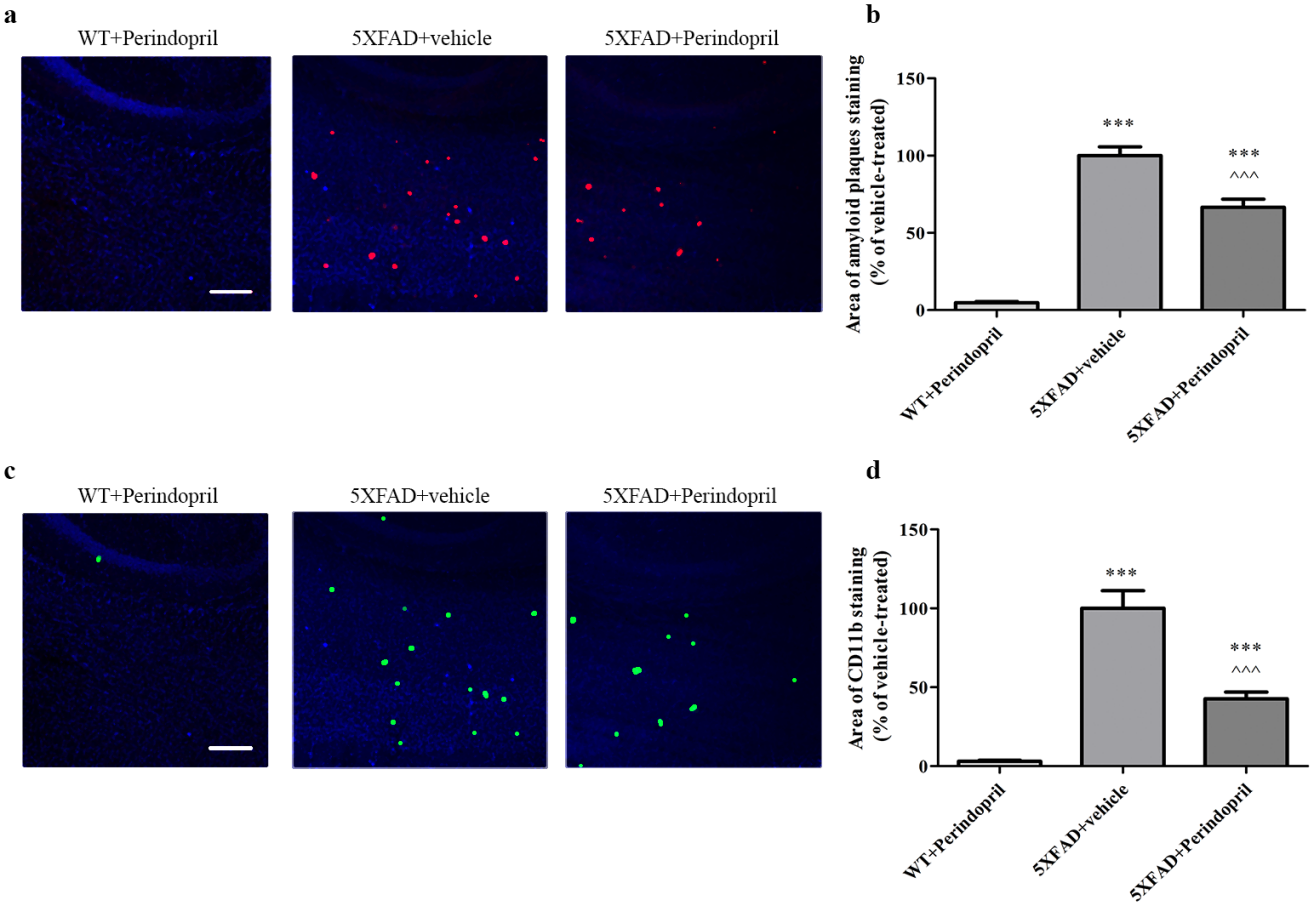
Intranasal administration of perindopril decreases amyloid plaques and CD11b staining in the cortex of 3-month old 5XFAD mice. Mice were treated with perindopril or vehicle (saline) for 3.5 weeks. The brains of 3-month-old mice were sectioned and immunolabeled with anti-Aβ (red) and anti-CD11b (green) antibodies and countersained with DAPI (blue). **(a, c)** Representative brain section of WT or 5XFAD mice treated with 1 mg/kg/day perindopril or with vehicle. Each experiment included 5 mice per group (n = 15 in total). (**b, d)** Quantification of the average sum of Aβ-stained area **(b)** or of CD11b-stained area **(d)**, are represented as the mean ± SEM percentage of stained area in the corresponding vehicle-treated group in at least 3 determinants. Statistical significance was determined using one-way ANOVA, followed by a Tukey—Kramer Multiple Comparison Test. **P<0.01 vs. WT+perindopril; ***P<0.001 vs. WT+perindopril; ^^^P<0.001 vs. 5XFAD+vehicle. Scale bar is 200 μm.

As compared with the vehicle-treated group, 3.5 weeks intranasal treatment with the ACEI perindopril (1 mg/kg/day) resulted in a reduction of 35% and 55% in amyloid plaques and in CD11b staining, respectively, in the cortex of 3-month-old 5XFAD mice (Fig 6). Neither amyloid plaques nor CD11b staining appeared in the cortex of age-matched WT mice.

## Discussion

In the present study we show a long term anti-inflammatory effect of telmisartan manifested by attenuation of LPS-induced NO microglial cell line as well as in primary cultures. TNF-α and IL1-β production as well as and iNOS protein expression induced by LPS were decreased by telmisartan in BV2 microglia. In glial cells, LPS is known as a potent stimulator of the production of proinflammatory mediators and reactive oxygen species. As shown by Miyoshi et al, LPS-induced primary rat microglia cultures also displayed elevated expression of AngII protein and increased AT_1_R mRNA levels [27]. Ang II itself was identified as paracrine mediator of sustaining brain inflammation in neurodegenerative disease models [48]. Taken together, these findings suggest that LPS and AngII may lead to increased production of various proinflammatory factors [7], including TNF-α, IL1-β and NO, which cohesively activate microglia, astrocytes and inflammatory cascades [49,50].

The reduction of LPS-induced NO, IL1-β and TNF-α levels by telmisartan suggests a brain modulatory role for the latter since these pro-inflammatory mediators stimulate the immune response and aggravate the neurodegradative state. [51,52]. The attenuation of TNF-α protein and IL1-β mRNA levels in LPS-stimulated microglia, as reported by Xu Yuan et al., was observed after short term (2 h) pre-treatment with telmisartan [53]. In the present study, a 24 h-inhibitory effect of telmisartan on microglial TNF-α, IL1-β and NO production was shown, suggesting changes in the levels of proteins responsible for the synthesis of NO, TNF-α and IL1-β. Indeed, Western blot analysis for iNOS showed that 24h-treatment with telmisartan decreased iNOS protein levels by 65–85% in LPS-stimulated cells.

Telmisartan and perindopril (1–3 mg/kg/day), upon systemic administration, were shown to penetrate the brain to various extents in different animal models [47,54,55]. Noda A et al [47] also showed blockade of AT_1_R following telmisartan penetration into the rhesus macaques brain. Oral administration of perindopril (1 mg/kg/day) showed significant inhibitory effect (by 50%) on brain ACE activity [31].

For *in vivo* studies, the 5XFAD mouse model was applied. This model is unique in that the mice develop rapid and extensive Aβ deposits alongside gliosis, which begins from 2-month of age. Plaques initially accumulate in the cortex, and expand to the hippocampus as the mice age. Moreover, the brains of 5XFAD mice exhibit neuroinflammation and gliosis, which are proportional to the levels and production period of the amyloid plaques [38]. As the 5XFAD mouse model robustly develops amyloid plaques compared with other AD mouse models [38], it presents a greater challenge for drugs to modulate plaque formation and gliosis. Our *in vivo* results also suggest an anti-inflammatory and beneficial role of intranasally administered telmisartan (ARB) and perindopril (ACEI), which attenuated Aβ plaques burden and microglia/macrophages activation in 5XFAD mice [35].

Telmisartan was reported to have a protective effect on cognitive impairment in an AD mouse model injected intracerebrally with the Aβ peptide [33]. Consistent with this, recent human prospective cohort [56] and nested case control [57] analyses reported a lower incidence and a slower rate of progression to dementia and AD in patients prescribed ARBs. Similarly, studies in young Aβ-injected mice [32,33,58] or adult Tg2576 AD mice [36] have shown the ability of ARBs to improve cognition. In contrast, AngII inhibition by ARBs in young or aged triple-transgenic AD mice indicated no benefit on the amyloid, tau or cognitive pathology [59,60].

It is controversial whether ACEI can exert beneficial effects on cognitive decline or AD. Among other studies, the Perindopril Protection Against Recurrent Stroke study [61] has demonstrated that antihypertensive treatment based on perindopril, a brain-penetrating ACEI, can reduce cognitive decline in patients with cerebrovascular disease. Furthermore, it has been suggested that brain penetrating ACEI can reduce the incidence of AD in elderly hypertensive patients [62–64]. However, the speculated role of ACE as an Aβ-degrading enzyme raised the concern that ACEI treatment may enhance Aβ_1–42_ levels in the brain of AD patients [65]. As such, the impact of inhibiting ACE on the accumulation of amyloid in AD patients remains unclear. Indeed, several studies have shown that the ACEI captopril promoted Aβ accumulation in the media of cells expressing human APP, and Aβ_1–42_ depositions in the brain of an AD mouse model [65,66]. Other studies reported that the administration of ACEIs, such as captopril, perindopril, or ramipril, failed to show such an effect and did not elicit changes in brain Aβ levels in APP-treated transgenic mice [31,67] or in a clinical trial [68].

Our findings show a reduction in Aβ deposition in specific brain areas of 5XFAD mice treated intranasally with perindopril. This effect may be attributed to the intranasal delivery, which was not tested previously with perindopril. Intranasal delivery of ARBs and ACEI was indeed expected to increase the direct effects of these compounds in the brain. [35]. As a matter of fact, chronic intranasal treatment with an ARB (losartan) dramatically decreased plaque number in the APP/PS1 AD mouse model [69]. Moreover, in clinical trials with AD patients, nasal application of insulin showed improvement in memory tasks and CSF biomarkers [70].

The removal of Aβ plaques can be achieved by activated microglia, which phagocyte fibrillar Aβ [71], or by an enzymatic degradation of the Aβ peptide, [72]. *In vitro* and *in vivo* studies suggest that the neutral endopeptidase neprilysin (NEP), the insulin-degrading enzyme (IDE), and the endothelin-converting enzyme-1 (ECE-1) are, potentially, Aβ-degrading enzymes [73,74]. In AD mouse models, NEP or IDE overexpression prevented the amyloid plaque pathology and premature death [75], and downregulation of NEP mRNA levels was reported in the cerebral cortex and in the hippocampus upon aging and in AD, even in the early stage of the disease [74]. Interestingly, Telmisartan can activate peroxisome- proliferator- activated receptor γ (PPARγ) and the latter serves as receptor-mediated mechanism for pharmacological upregulation of NEP [76].

In light of these findings, the positive effects of telmisartan and perindopril on amyloid plaques burden, as reported in the current study, may be attributed to possible up-regulation of Aβ-degrading enzymes. Moreover, since telmisartan is an ARB with potential strong PPAR-γ agonist properties [7,22,77], it is hypothesized that the effect observed *in vitro* and *in vivo* may be attributed not only to its AT_1_R antagonist characteristics but also to the activation of PPAR-γ. Prevention of cognitive decline due to PPAR-γ activation by telmisartan was shown by Mogi M et al [32] in AD mice. In addition, an interaction between PPAR-γ activation and AT_1_R expression was suggested by several studies [78,79]. To conclude, we provide evidence that a non-invasive intranasal delivery of telmisartan or perindopril may serve as an efficient alternative for their systemic administration, as it results in the attenuation of microglia/macrophages activation and in the reduction of Aβ plaques. Further studies are required to test whether an up-regulation of Aβ-degrading enzymes or an increased phagocytosis by activated microglia underlie the beneficial effects of telmisartan and perindopril.
